# Oct4 regulates DNA methyltransferase 1 transcription by direct binding of the regulatory element

**DOI:** 10.1186/s11658-018-0104-2

**Published:** 2018-08-16

**Authors:** Fengrui Wu, Qingqing Wu, Dengkun Li, Yuan Zhang, Rong Wang, Yong Liu, Wenyong Li

**Affiliations:** 1 Anhui Province Key Laboratory of Embryo Development and Reproductive Regulation, Fuyang Normal University, Fuyang, China; 2 Anhui Province Key Laboratory of Environmental Hormone and Reproduction, Fuyang Normal University, Fuyang, China

**Keywords:** *Dnmt1*, Oct4, Transcription regulation, Kunming white mouse

## Abstract

**Background:**

The transcription factor Oct4 plays a pivotal role in the pre-implantation development of the mouse embryo. DNA methyltransferase 1 (Dnmt1) maintains the changes in DNA methylation during mammalian early embryonic development. Little is known of the role of Oct4 in DNA methylation in mice. In this study, Kunming white mice were used as an animal model to reveal any correlation between DNA methylation and Oct4 during mammalian embryonic development.

**Results:**

The expressions of *Dnmt1* and *Oct4* were initially studied using real-time PCR. They exhibited different patterns during the pre-implantation stage. Moreover, by using a promoter assay and ChIP analysis, we found that the transcriptional activities of *Dnmt1* in mouse NIH/3 T3 cells and CCE cells were regulated by Oct4 through direct binding to the − 554 to − 294 fragment of the upstream regulation element of *Dnmt1*. The downregulation of Dnmt1 expression and enzyme activity by mouse Oct4 were further confirmed by transfecting *Oct4* siRNA into mouse CCE cells.

**Conclusion:**

Our results indicate that Oct4 is involved in DNA methylation through the regulation of *Dnmt1* transcription, especially during the early stages of mouse pre-implantation embryo development.

**Electronic supplementary material:**

The online version of this article (10.1186/s11658-018-0104-2) contains supplementary material, which is available to authorized users.

## Background

The transcription factors Oct4, Sox2, Klf4 and c-Myc (referred to as the OSKM factors or Yamanaka factors) are essential for mammalian embryo development, especially in the embryonic pre-implantation stage [1]. A previous study found that the activities of these factors are essential for the self-renewal and pluripotency of mammalian embryonic stem cells (ESCs) and adult stem cells [2].

Recently, a novel type of stem cells called induced pluripotent stem (iPS) cells was successfully generated through retroviral introduction of the genes for the critically important OSKM factors. The highest efficiencies of induced pluripotency were achieved with combinations of all four factors [3].

Although little information is available on the molecular biology events that occur during embryonic pre-implantation and further development, there is sufficient evidence to indicate that these four factors have important functions in maintaining pluripotency and differentiation potential [1]. However, many studies have found that adult mouse neural stem cells and human fetal neural stem cells can be directly reprogrammed to iPS cells through ectopic expression of Oct4 alone, suggesting that Oct4 is required and sufficient to reprogram mammalian somatic cells to pluripotency [4, 5].

Oct4 has been demonstrated to be a key transcription factor controlling pre-implantation development in the mouse embryo [1]. Notably, Oct4 kinetics was identified as a predictive measure of developmental cell lineage patterning in the early mouse embryo [6]. Furthermore, the specification of pluripotent cell identity requires the embryonic genome to express Oct4 during mouse development [7]. In mouse blastocysts, Oct4 is required for the expression of multiple epiblast and primitive endoderm genes, and for the operation of multiple metabolic pathways essential for the continued growth of the pre-implantation embryo [8]. These findings suggest that Oct4 has crucial roles in the early stages of development and differentiation, as evidenced elsewhere [9].

DNA methylation is an important epigenetic modification event during mouse embryonic development and in the processes of somatic cell reprogramming and gene silencing [10–12]. Dnmt1 is the major DNA methyltransferase responsible for methylating hemi-methylated cytosines in CpG sequences. It also acts as a maintenance methyltransferase that maintains genome-wide methylation patterns during genomic DNA replication [13, 14]. Many studies have shown that Dnmt1 plays a crucial role in normal mammalian development, and in cell proliferation and survival [15]. Mutation of *Dnmt1* results in extensive demethylation of the genome DNA, embryonic lethality, loss of imprinting, and alterations in X chromosome inactivation during mouse embryonic development, while the absence of Dnmt1 leads to the death of ES cells [16]. *Dnmt1* knockdown in germline cells leads to their immediate apoptosis [17].

During mouse oogenesis and pre-implantation development, Dnmt1 transcripts and protein were found to be expressed and proven to be responsible for the maintenance of methylation during pre-implantation stages other than the eight-cell embryo [18].

The expression of Oct4 and DNA methylation have been shown to be critical in the development of the mammalian pre-implantation embryo and in cell reprogramming [19]. However, the results on the correlation between mouse Oct4 and Dnmt1 in these processes are still contradictory. Here, we used Kunming mice as a model to examine the expressions of *Dnmt1* and *Oct4* during the pre-implantation stage. The luciferase promoter assay and chromatin immunoprecipitation (ChIP) were performed to investigate the binding of Oct4 to the cis-regulation element of *Dnmt1*. RNAi of *Oct4* in mouse CCE cells was also carried out to examine whether the expression level and enzyme activity of Dnmt1 was regulated by Oct4 in vitro. Our results are consistent with those in an earlier report [20]. Thus, we have the reason to believe that Oct4 is involved in DNA methylation through regulation of the transcription of *Dnmt1* and that this would also be true for human mesenchymal stem cells.

## Results

### Mouse *Dnmt1* and *Oct4* expression in pre-implantation embryos from the zygote stage

**Fig. 1:**
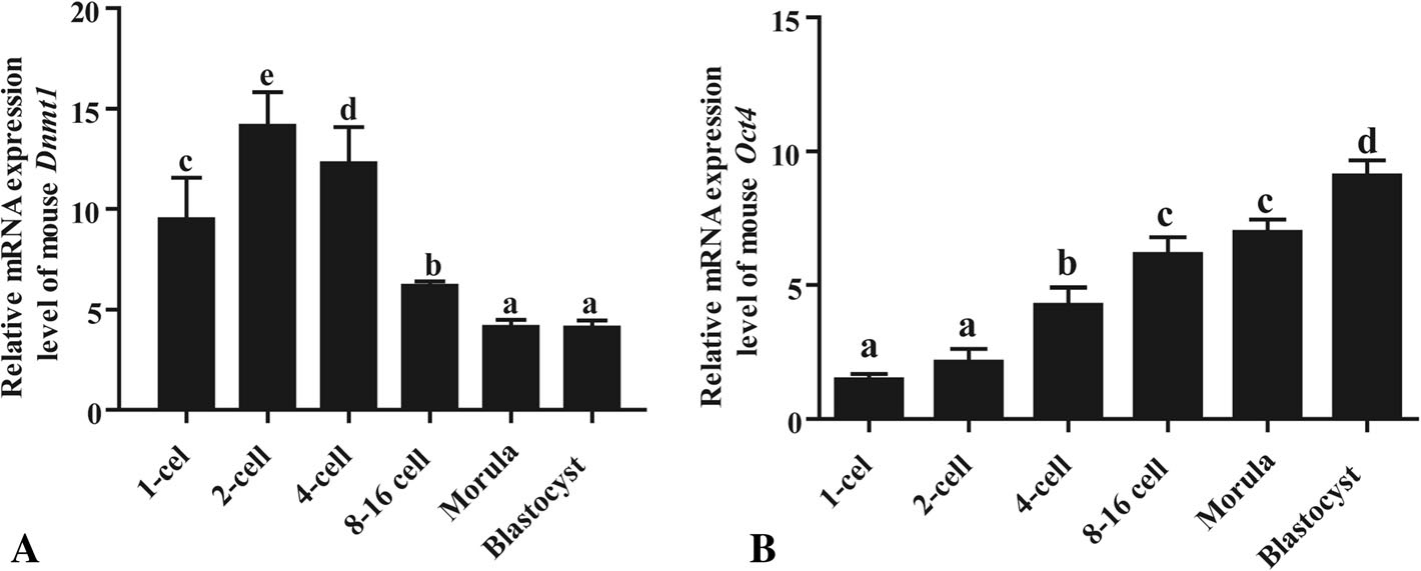
The expression pattern of mouse *Dnmt1* (**a**) and *Oct4* (**b**) in pre-implantation embryos, as detected using real-time PCR. Data are expressed as the means ± SE of three replicates, and the values sharing the same letters are not significantly different at *p* < 0.05

Quantitative PCR results showed that the expression level of mouse *Dnmt1* in the embryos increased from the zygote to the 4-cell stage and dramatically decreased from the 8-cell stage onwards (Fig. 1a), whereas the level of *Oct4* increased steadily during the pre-implantation embryo stage (Fig. 1b).

### Oct4 regulates mouse Dnmt1 gene transcription in vitro

Based on a prediction made using TFSEARCH and PROMO, a potential Oct4-binding site(TTTTGCAT/ATGCAAAA) was found in the − 475 to − 468 bp region relative to the transcription start site (TSS). To confirm the potential Oct4-binding site, four luciferase reporters were constructed and transfected in two different cell lines.

**Fig. 2:**
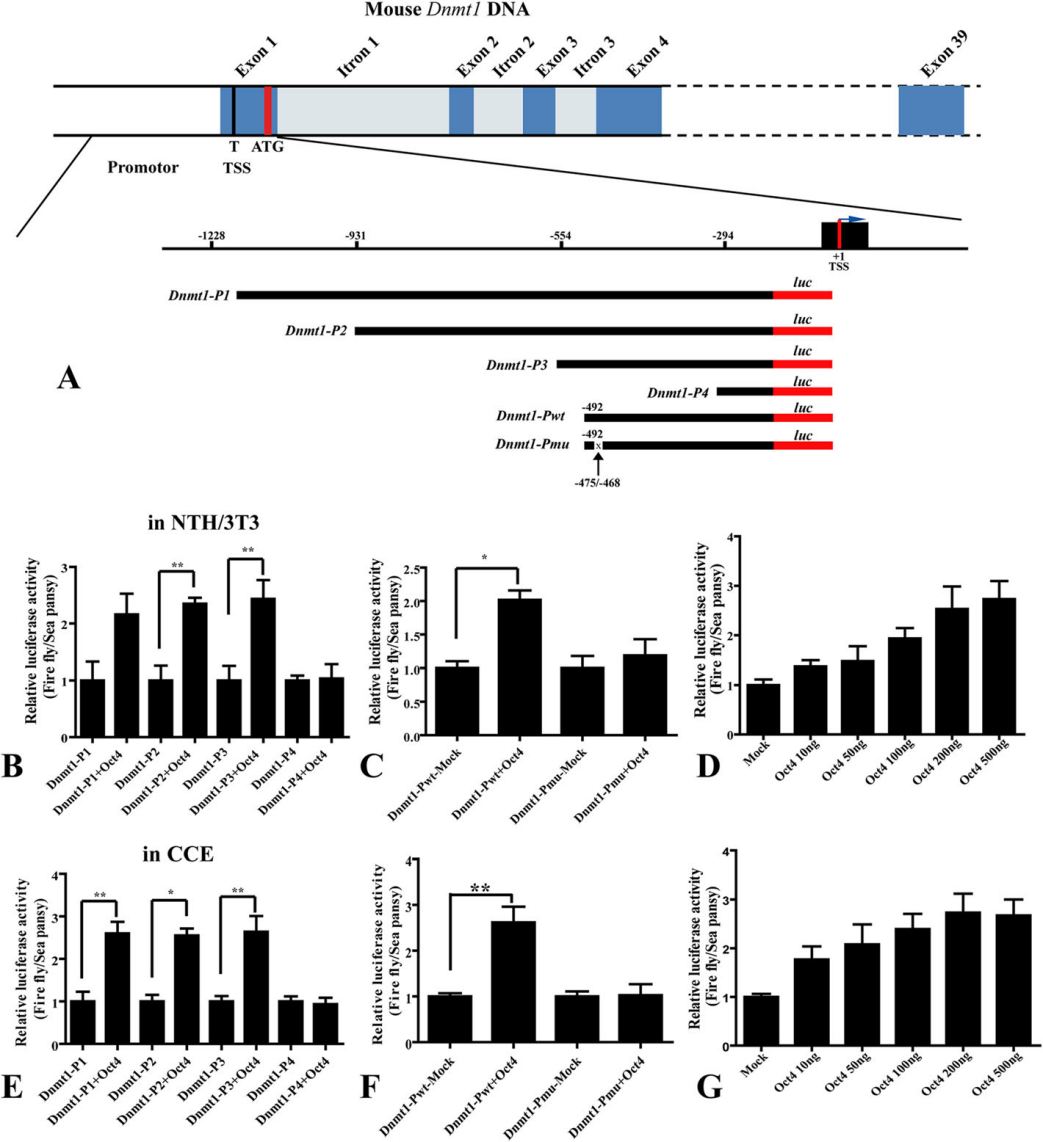
Promoter analysis of mouse Dnmt1 in NIH3T3 and CCE cells using the luciferase assay. **a** Schematic structures of mouse *Dnmt1* gene constructs used in this study. **b**, **c**, **e** and **f** – Oct4 enhanced the promoter activity of mouse Dnmt1. *Oct4*-pcDNA3.1 (100 ng) plasmid was co-transfected with a series of sequential deletion constructs (Dnmt1 P1–4) and a mutant of the mouse Dnmt1 promoter (500 ng/well) into NIH3T3 and CCE cells. **d** and **g** 10, 50, 100, 200, and 500 ng of *Oct4*-pcDNA3.1 expression plasmid was co-transfected with mouse Dnmt1-P3 promoter (500 ng/well) into NIH3T3 cells (**d**) and CCE cells (**g**). The total amount of the transfected plasmid, including the pRL-TK control vector (100 ng/well), was adjusted to 1.0 μg with pcDNA3.1 empty vectors. Firefly and *Renilla luciferase* activities were measured 48 h after the transfection. The relative luciferase activity was calculated by dividing the activity of firefly luciferase by the activity of *Renilla* luciferase. The data are presented as the means ± SD for triplicate transfections

The results showed that the relative luciferase activities were higher in the groups that were co-transfected with Dnmt1-P1, 2 and 3 and Oct4 than in the separate control (Fig. 2a, b and e). Deletion of the upstream region from − 1228 to − 554 bp had no effect on the activation mediated by Oct4 (Fig. 2b and e), indicating that the first three constructs (Dnmt1-P1–1 through − 3) did not alter the Oct4 effect on the transcriptional activity of Dnmt1. By contrast, a decline in the relative luciferase activity in the shortest *Dnmt1* promoter (-P1–4) was detected, indicating that the Oct4 positive regulatory element in the region between − 554 and − 294 bp relative to TSS could be lacking.

These findings are consistent with the results predicted using the web tools. As expected, mutation of this element to CCCCATGC remarkably decreased the promotor activity of *Dnmt1* and the transcription activation efficiency of Oct4 (Fig. 2c and f). This result suggests that TTTTGCAT could be the possible binding site for Oct4 in the mouse *Dnmt1* promoter region. Notably, co-transfection of Dnmt1-P1–3 and Oct4 increased luciferase activity in a dose-dependent manner (Fig. 2d and g).

### Oct4 direct binding to the regulatory elements of mouse Dnmt1

**Fig. 3:**
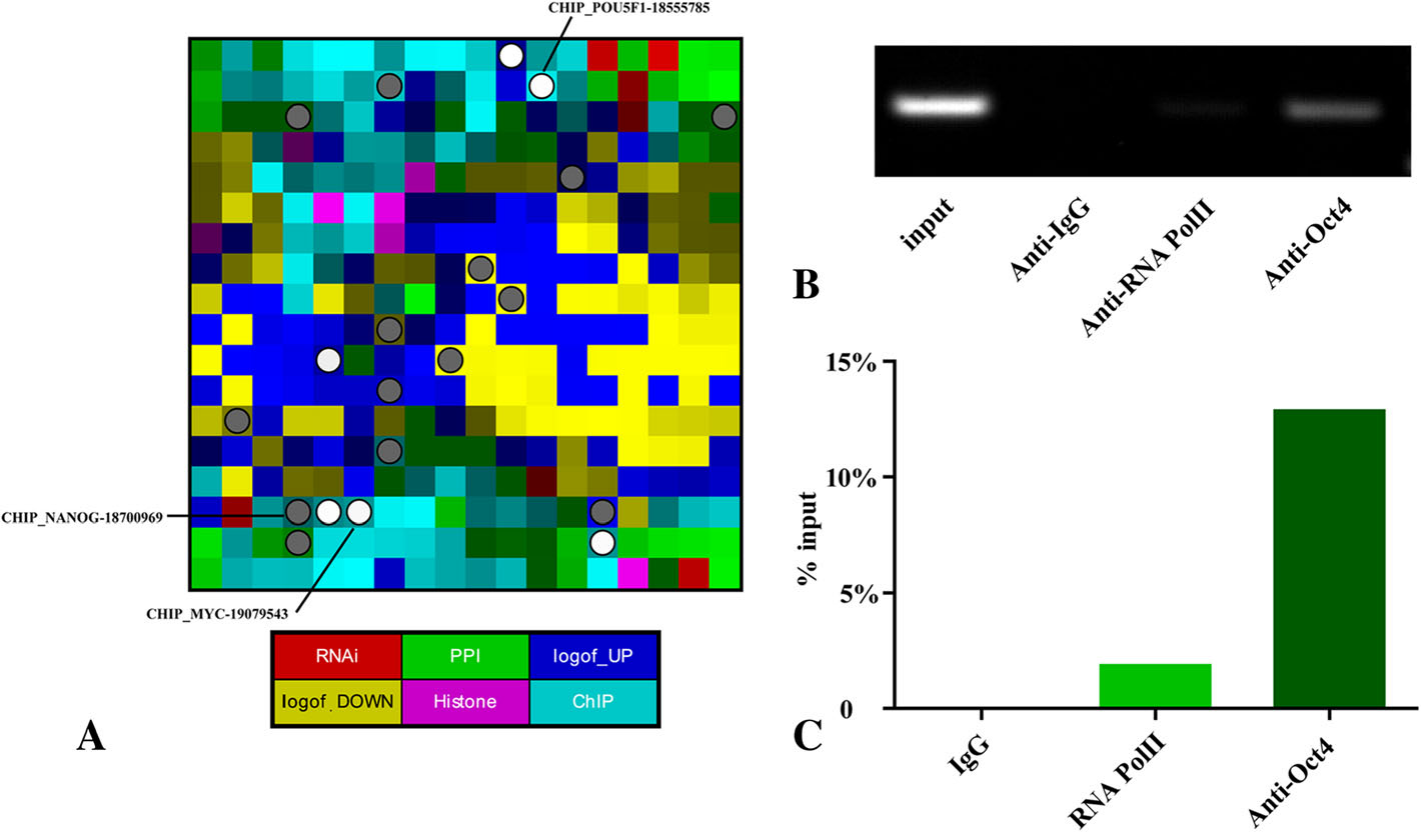
The chromatin immunoprecipitation assay was conducted to confirm the binding of Oct4 to the promoter of mouse Dnmt1 in vivo. **a** Enrichment results for Dnmt1 determined via ChIP-Seq. The different colors indicate the experiment type, and each square represents a list. Enriched terms are highlighted in circles and three terms are annotated. The brightness indicates the level of local similarity between the lists. **b** Anti-RNA Pol II and anti-Oct4 antibodies precipitated proteins bound in vivo to the specific amplified sequence of the mouse Dnmt1 promoter (the region between − 554 and − 294 bp). Non-specific IgG (negative control antibody) failed to do. A 213-bp length of PCR products were resolved on 1.5% agarose gel and stained with ethidium bromide to visualize the bands. **c** Analysis of the Dnmt1 promoter physically associated with Dnmt1 using ChIP-qPCR assay in the CCE cells

Next, we examined whether Oct4 binds to the *Dnmt1* promoter in vitro. The ESCAPE database was queried with the gene name Dnmt1 to conduct the enrichment analysis. As shown in Fig. 3a, CHIP_POU5F1–18,555,785 was markedly enriched (*p* = 2.167e-2). Additionally, to associate the Oct4-binding siteinformation in the *Dnmt1* promoter, a high association score was obtained for Oct4 (0.955676) based on the relative distance to the TSS of *Dnmt1*, indicating that *Dnmt1* is one of the potential target genes for Oct4 in human and mouse embryonic stem cells.

The ChIP assay was employed to investigate the binding affinity. The chromatin (from CCE cells) was immunoprecipitated with anti-RNA Pol II antibody (as a positive control), anti-IgG antibody (as a negative control), and a specific antibody against mouse Oct4. As shown in Fig. 3b and c, the anti-RNA Pol II and anti-Oct4 antibodies precipitated the proteins bound in vivo to the specific amplified sequence of the mouse Dnmt1 promoter in the region between − 554 and − 294 bp. Conversely, the non-specific IgG antibody failed to precipitate in vivo the proteins bound to this sequence, suggesting that mouse Oct4 has the potential to bind to the fragment of Dnmt1.

### Dnmt1 expression was downregulated by Oct4 RNAi in CCE cells

**Fig. 4:**
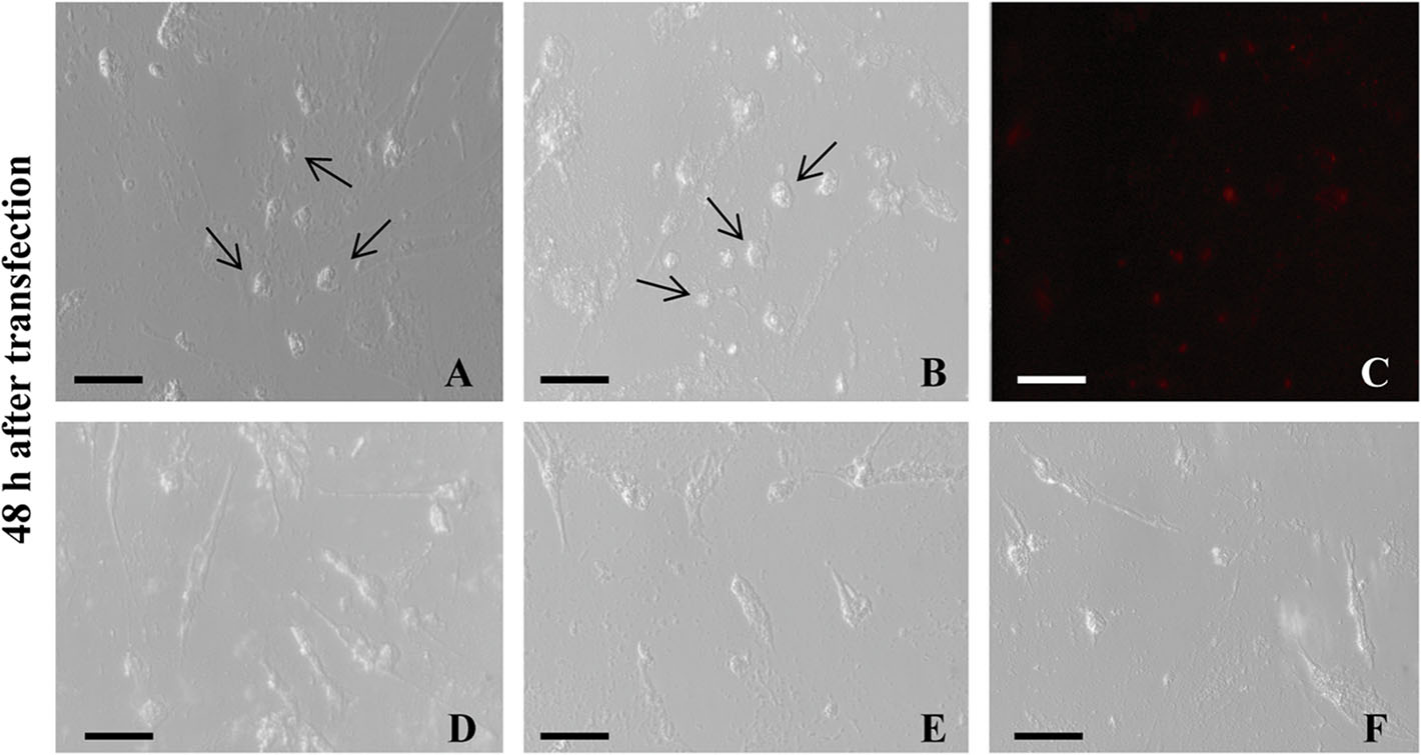
The effect of mouse Oct4 knockdown in CCE cells. **a** and **b** CCE cells were transfected without (**a**) or with (**b**) the negative control alone for 48 h. **c** More than 85% of the cells were successfully transfected with Alexa Fluor Red Fluorescent Oligo. **d**, **e** and **f** The self-renewal and undifferentiated state of CCE cells were not maintained when transfected with the three siRNA of Oct4 – R1 (**d**), R2 (**e**) and R3 (**f**) when tested 48 h after the transfection. The arrows indicate the normal CCE mouse ES cells. The images were photographed with Leica DMI3000B at 200× magnification. Scale bar = 20 μm

We investigated whether the knockdown of Oct4 in CCE cells can decrease the mRNA level and enzyme activity of Dnmt1. To determine the transfection efficiency, 20 nM Red Fluorescent Oligo was transfected into CCE cells. As shown in Fig. 4a–c, the efficiency reached > 85%, indicating that these conditions could be used to perform the subsequent experiments. Notably, the morphology of CCE cells was obviously changed 48 h after transfection with the three siRNA of *Oct4* (R1, R2, and R3; Fig. 4d–f), indicating that the self-renewal and undifferentiated state of the CCE cells were not maintained.

**Fig. 5:**
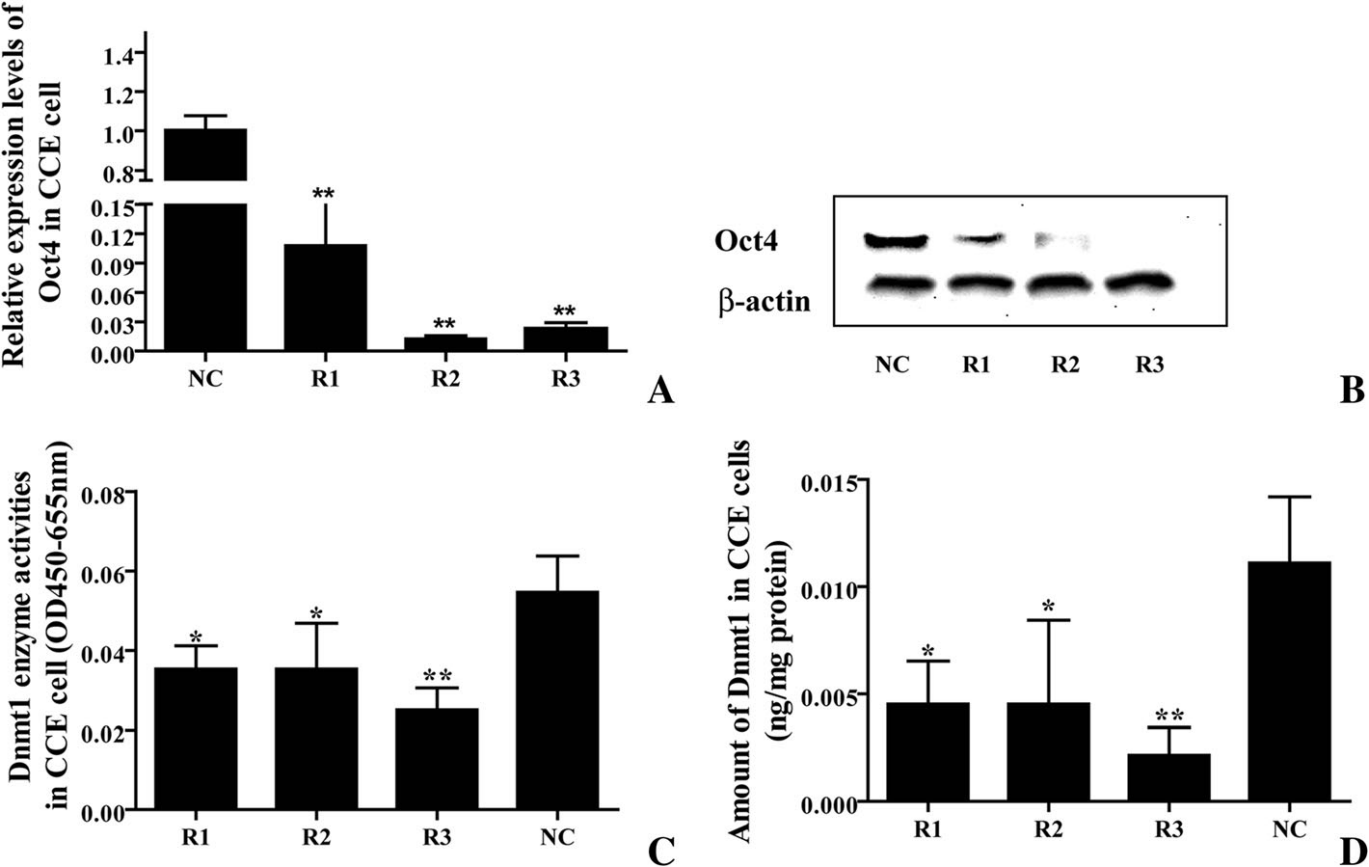
The Dnmt1 assay showed the decrease in Dnmt1 in CCE cells transfected with mouse Oct4 siRNA. **a** and **b** The mRNA (**a**) and protein (**b**) levels of Oct4 were downregulated by the three mouse Oct4 siRNA. **c** and **d** The final OD level (equal to the 450 nm OD minus the 655 nm OD) (**c**) and the amount of Dnmt1 (**d**) were reduced in mouse Oct4 siRNA R1-, R2-, and R3-transfected groups when compared with the negative control. Results are presented as the means ± SD. The final ODs from triplicate transfected samples were measured using microplate reader. * and **Statistically significant differences obtained with Student’s *t*-test for comparison with the negative control at *p* < 0.05 and 0.01

Real-time PCR and western blot were used to measure the mRNA and protein levels of *Oct4* in the siRNA-transfected CCE cells. The *Oct4* mRNA and protein levels in R1-, R2-, and R3-transfected groups dramatically decreased, while those in the control remained unchanged (Fig. 5a and b). The results show that both R2 and R3 reduced *Oct4* mRNA by 95% of the level for the blank control at 48 h post-transfection. The protein levels of Oct4 were also obviously reduced in the R2- and R3-transfected groups (Fig. 5a and b).

A Dnmt1 assay was also performed to detect the enzyme activity of Dnmt1 in the transfected siRNA CCE cells. The value of the final OD (equal to the OD at 450 nm minus the OD at 655 nm) of the R2- and R3-transfected groups were 1.5 to 2 times lower than those of the negative control. The change rates and the amounts of Dnmt1 in the three siRNA CCE were approximately 60–80% of those of the negative control (Fig. 5c and d). These results indicate that the decline in Dnmt1 activity is dependent on the function of Oct4 in CCE cells.

## Discussion

Kunming white mice are widely used as model animals in China. The dynamic expression and cellular localization of Dnmt1 and Oct4 in pre-implantation embryos of other mouse strains have been reported [1, 21], but little is known about the mRNA expression pattern of *Dnmt1* and *Oct4* in pre-implantation embryos of the Kunming white mouse.

It is well known that epigenetic modification of the genome plays important roles in mouse early development. Many studies have shown that the global DNA methylation levels gradually decrease from 1-cell to 16-cell or morula stage embryos [22], but reaching their highest levels in blastocysts by activating the de novo methylation process on top of the normal maintenance methylation [23].

In this study, we found that *Dnmt1* levels are inversely related to *Oct4* mRNA levels during pre-implantation embryo stages. Our results show that the mRNA level of *Dnmt1* before the 4-cell stage was relatively higher than that from the 8-cell stage onwards, further indicating that Dnmt1 is a maintenance methyltransferase in the pre-implantation period [14, 24]. The genomic re-methylation in the inner cell mass (ICM) and hypomethylated state in the trophectoderm of the blastocyst are achieved passively by decreasing the expression of *Dnmt1* or caused by de novo methyltransferases (*Dnmt3a* and *Dnmt3b*) [25]. This expression pattern of *Dnmt1* also responds to the global DNA methylation levels during the mouse pre-implantation stage. Furthermore, while initially present as a maternal transcription factor in the oocyte [26], the *Oct4* level is gradually raised by the embryo throughout the pre-implantation period and correlates with an undifferentiated phenotype. This indicates that Oct4 could function as a key player and construct gene regulation networks with other genes during mouse early development.

In a previous study, Oct4 was observed to be located in the ICM cells of blastocysts [27]. It is worth noting that in the Kunming white mouse, the mRNA expression level of *Oct4* increases with embryonic development and peaks during the blastocyst stage, as has also been reported in other mouse strains [1]. Inspired by these previous and present data, we speculated that some correlation may exist between Oct4 and the transcription of *Dnmt1*.

Using bioinformatic analysis, an octamer motif was predicted in the region between − 475 and − 468 bp relative to TSS. Therefore, promoter analysis was performed to investigate whether *Dnmt1* transcription is regulated by Oct4. Sequential deletion promoters of mouse Dnmt1 were isolated and cloned into luciferase reporter vectors, including a luciferase plasmid bearing a mutation in the − 475 to − 468 bp Oct4 binding motif. Using transfection assays, we found that no significant difference in the luciferase activities of mouse *Dnmt1* among the Dnmt1 P-1 to P-3 groups when co-transfected with mouse Oct4, but significant differences were observed between those groups and the respective control treatments. On the other hand, the luciferase activity of Dnmt1 P-4 was obviously lower than those of Dnmt1 P-1 to − 3 groups, indicating that there is a cis-regulatory element of Oct4 in the mouse *Dnmt1* promoter region (the region from − 554 to − 294 bp). Because of the endogenous high expression of Oct4 in CCE cells, we found that their luciferase basal activity was higher than that for NIH cells without overexpression of external Oct4 when co-transfected with mouse Dnmt1-P1 promoter. Meanwhile, the luciferase activity in CCE cells was lower than that for control cells (Dnmt1-Pwt promoter) when co-transfected with mouse Dnmt1-Pmu promoter, but there was no significant difference between Dnmt1-Pmu promoter-transfected groups with or without overexpression of external Oct4 (Additional file 1: Figure S1). These data indicate that mouse *Dnmt1* gene expression might be regulated by Oct4 through direct binding to the promoter region of Dnmt1.

During MSC proliferation, *Dnmt1* was upregulated by Oct4 through direct binding to its promoter, leading to decreased expression of p16 and p21 and the genes associated with their development and lineage differentiation [20]. Breast cancer-associated gene1 (BRCA1) can bind to the *DNMT1* promoter through a potential OCT1 site in both mouse and human cells [28]. During cell transformation and tumorigenesis, mouse *Dnmt1* transcription is regulated through both E2F-Rb-HDAC-dependent and -independent pathways [29]. We employed the ChIP assay and sensitive two-color EMSA assay (Additional file 2: Figure S2) to confirm that the DNA fragment between − 554 and − 294 bp of *Dnmt1* was directly bound by Oct4 in vitro. These results were consistent with those from other studies [30, 31].

Furthermore, in our study, silencing of Oct4 expression significantly reduced the expression and enzyme activity of Dnmt1 in CCE cells, while the overexpression of *Oct4* obviously increased the amount of Dnmt1 in NCI-H157 cells (a line of human non-small cell lung cancer cells with a high endogenous Dnmt1mRNA level; Additional file 3: Figure S3).

These abovementioned previously reported findings, together with our data, reveal that *Dnmt1* gene expression in vivo is regulated by Oct4. However, the DNA methylation status of the mouse *Oct4* gene upstream region has been considered essential for its gene expression, i.e., the *Oct4* enhancer/promoter region was hypomethylated in ES cells and the expression of *Oct4* mRNA was detected in the Dnmt1^n/n^ placenta but not in the wild-type placenta [32]. In addition, aberrant *Oct4* gene expression was identified in another examination [19]. These results indicate that the maintenance of DNA methylation status by Dnmt1 in mice might be accompanied with a change in the expression level of *Oct4* mRNA. Based on these results, we have reason to believe that DNA methylation catalyzed by Dnmt1 is modulated through Dnmt1 expression, which regulated by Oct4, whereas the spatial and temporal profiles of Oct4 are influenced through the DNA methylation status of its promoter and DNA methylation-mediated gene silencing.

## Methods

### Animals and collection of embryos

All animals were maintained with a photoperiod of 14 h light and 10 h dark at 20–25 °C for at least 2 weeks before use. Zygotes, 2-cell, 4-cell and 8-cell embryos, morulae, and blastocysts were collected for this study as described previously [33, 34]. All animal studies were approved by the Institutional Animal Care and Use Committee at Fuyang Teachers College in Anhui Province, China. This study was conducted in strict accordance with the recommendations in the 1988 Regulations on the Management of Laboratory Animals in China. The Kunming white mice that were used as a model in this examination were purchased from the Experimental Animal Center of Anhui Medical University (Certification of quality #34000200000077, 34,000,200,000,078).

### RNA extraction and real-time PCR

Fifty embryos were used for each time point, and three replicates were performed for each stage. Total RNA extraction, cDNA synthesis from all the samples, and real-time PCR were conducted according to the manufacturer’s instructions as described previously [33]. *Gapdh* was used as an internal control. The threshold cycle (Ct) was defined as the fractional cycle number by the method of global minimum. The ratio change in *Oct4* and *Dnmt1* relative to the *Gapdh* control gene was determined using the 2^-△△Ct^ method [33]. Data are expressed as the means ± SE for the three replicates.

A Kruskal-Wallis test, which was conducted with GraphPad Prism 5 software (GraphPad Software), was used to determine if there was a significant difference between the means (*p* < 0.05). All primers used for the study are listed in Additional file 4: Table S1.

### Plasmid constructs

The 1228-, 931-, 554- and 294-bp fragments of the 5′-flanking regions of the mouse *Dnmt1* gene were generated via PCR and subcloned into the pGL3-Basic Vector (Promega Corp.) within the *Mlu* I and *Hind* III sites. The software Promoter 2.0 Prediction Server (http://diyhpl.us/~bryan/irc/protocol-online/protocol-cache/TFSEARCH.html) was used to predict the transcription start sites of mouse *Dnmt1*. Luciferase plasmid bearing a mutation in the − 475 to − 468 bp Oct4-binding motif was constructed via PCR-mediated mutagenesis using primers containing the mutations. The software TFSEARCH ver. 1.3. (http://diyhpl.us/~bryan/irc/protocol-online/protocol-cache/TFSEARCH.html) and PROMO searching tools (http://alggen.lsi.upc.es/) were employed to analyze all possible binding sites on the sense and antisense chains of the mouse *Dnmt1* promoter. Mouse *Oct4* were amplified and cloned into the pcDNA3.1 expression vector (Invitrogen) using gene-specific open reading frame (ORF) primers as described in our previous study [33]. All inserted sequences were further confirmed via sequencing by Life Technologies Corporation.

### Cell culture and luciferase assay

Using Lipofectamine 2000 (Invitrogen), mouse fibroblast cell line NIH/3 T3 cells were transfected with the following plasmids:

1. 500 ng of normal or truncated constructs or mutants of the mouse *Dnmt1* promoter, which were cloned into the pGL3-Basic luciferase reporter vector;
2. 10, 50, 100, 200 or 500 ng of *Oct4*-pcDNA3.1 expression plasmid;
3. pRL-TK (Promega) at 100 ng/well.

*Renilla* luciferase from pRL-TK was employed as an internal control for transfection efficiency. Firefly luciferase and *Renilla* luciferase readings were obtained using the Dual-Luciferase Reporter Assay System (Promega) and GloMax 20/20 Luminometer (Promega). Cell culture, transient transfection and luciferase assays were performed as reported previously [33]. For CCE mES cells, a mESC line derived from the 129/Sv mouse strain, donated by Professor Sijin Liu (Research Center for Eco-Environmental Sciences, Chinese Academy of Sciences), was cultured and transfected with the same plasmids as the NTH/3 T3 cells using Effectene Transfection Reagent (Qiagen), as described previously [35, 36].

### Chromatin immunoprecipitation (ChIP) assay

The online program ESCAPE (http://www.maayanlab.net/ESCAPE) and the GEO database with ID number GSE11431 were used to confirm whether Oct4 binds to the Dnmt1 promoter in vitro as described previously [30, 31]. ChIP assays were also performed with the Imprint Chromatin Immunoprecipitation Kit (Cat. No. CHP1, Sigma) according to the manufacturer’s instructions. Briefly, CCE cells were cross-linked in 1% formaldehyde at room temperature for 10 min. The isolated nuclei were lysed and followed by solubilization with shearing buffer containing a protease inhibitor cocktail (Sigma). The chromatin was then sonicated and immunoprecipitated. The antibodies used for ChIP studies were anti-RNA Pol II antibody, anti-IgG antibody (provided in the kit) and anti-Oct4 antibody (Abcam, ab19857). After reverse cross-linking and DNA purification, DNA from input (1:10 diluted) or immunoprecipitated samples was assayed via PCR, and the products were separated using 1.5% agarose gel electrophoresis. For quantitative ChIP analysis, real-time PCR was carried out with SYBR Green PCR Master Mix as described above.

The Δ Ct value in the results uses the following formula:

$$ \Delta\ \mathrm{Ct}=\mathrm{Ct}\ \left(\mathrm{sample}\right)\hbox{-} \mathrm{Ct}\ \left(\mathrm{input}\right),\Delta\ \Delta\ \mathrm{Ct}\ \left(\Delta\ \mathrm{Ct}\ \mathrm{sample}\hbox{-} \Delta\ \mathrm{Ct}\ \mathrm{negative}\ \mathrm{control}\right) $$

The percent input was derived with:

$$ \Big({2}^{\left(\hbox{-} \Delta\ \Delta\ \mathrm{Ct}\ \mathrm{treatment}\right)}/{2}^{\left(\hbox{-} \Delta\ \Delta\ \mathrm{Ct}\ \mathrm{control}\right)}\;\mathrm{was}\ \mathrm{derived} $$

The primers used for ChIP analysis PCR reaction are shown in Additional file 4: Table S1.

### RNA interference (RNAi) and transfection

Lipofectamine RNAiMAX (Invitrogen Life Technologies) was used to transfect the Stealth RNAi siRNA against Oct4 into CCE cells. The Stealth RNAi Negative Control Duplexes (Invitrogen Life Technologies) were used as a negative control. BLOCK-It Alexa Fluor Red Fluorescent Oligo (Invitrogen) was used to facilitate the assessment and optimize the delivery of double-stranded RNA oligonucleotides into the CCE cells. These siRNA sequences were submitted to a BLAST search to ensure that only the mouse *Oct4* gene was targeted. The sequences of the three synthesized oligonucleotides were:

- R1 sense 5’-CCAAUGCCGUGAAGUUGGAGAAGGU-3′ and anti-sense 5’-ACCUUCUCCAACUUCACGGCAUUGG-3′;
- R2 sense, 5’-CCCGGAAGAGAAAGCGAACUAGCAU-3′ and anti-sense, 5’-AUGCUAGUUCGCUUUCUCUUCCGGG-3′;
- R3 sense, 5’-CCAAUCAGCUUGGGCUAGAGAAGGA-3′ and anti-sense, 5’-UCCUUCUCUUAGCCCAAGCUGAUUGG-3′.

RNAi transfection was conducted according to the manufacturer’s instructions. Gene knockdown assays were performed after the complexes were added to the cells and incubated for 48 h at 37 °C in a CO_2_ incubator.

### Gene knockdown and Dnmt1 assay

Real-time PCR and western blotting were conducted to investigate the mRNA and protein expression levels of mouse Oct4 and confirm whether Dnmt1 was downregulated. Total RNA extraction, cDNA synthesis, and real-time PCR were performed as described above. Total proteins were extracted from the RNAi-transfected CCE cells following the procedure detailed in the manual of the “NE-PER Nuclear and Cytoplasmic Extraction Reagents” (#78833, Thermo Fisher Scientific). The protein concentration was measured using a bicinchoninic acid assay on a NanoDrop 2000 spectrophotometer. Western blotting analysis was performed as described previously [37]. The antibody against mouse Dnmt1 was purchased from Abcam (ab13537). For the Dnmt1 assay, the RNAi-transfected CCE cells were initially lysed with NE-PER Nuclear and Cytoplasmic Extraction Reagent, and the EpiQuik DNMT1 assay kit (Epigentek, P-3011) was used to detect the amount of Dnmt1 according to the manufacturer’s instructions and as described previously [38].

## Conclusions

Our results demonstrate that Oct4 plays an important role in the transcription of *Dnmt1* by direct binding to a specific site on the *Dnmt1* promoter. The total amount of Dnmt1 in CCE cells is reduced by Oct4 as evidenced by the results of the knockdown assay. These findings might reveal a correlation between Oct4 and Dnmt1 during the stages of mouse pre-implantation embryo development and provide new insights into the mechanism of the early stages of mammalian embryonic development.

## Additional files

Additional file 1: **Figure S1.** Promoter analysis of mouse Dnmt1 in NIH3T3 and CCE cells using the luciferase assay. A – The promoter activity of mouse Dnmt1 in NIH3T3 and CCE cells. *Oct4*-pcDNA3.1 (100 ng) plasmid was co-transfected with Dnmt1-P1 into NIH3T3 and CCE cells. B – The promoter activity of mouse Dnmt1-Pwt and -Pmu in CCE cells. *Oct4*-pcDNA3.1 (100 ng) plasmid was co-transfected with Dnmt1-Pwt and -Pmu into CCE cells. The total amount of the transfected plasmid, including the pRL-TK control vector (100 ng/well), was adjusted to 1.0 μg with pcDNA3.1 empty vectors. Firefly and *Renilla luciferase* activities were measured 48 h after the transfection. The relative luciferase activity was calculated by dividing the activity of firefly luciferase by the activity of *Renilla* luciferase. The data are presented as the means ± SD for triplicate transfections. (TIF 306 kb)

Additional file 2: **Figure S2.** The results of the sensitive two-color EMSA assay showed direct binding of Oct4 to the mouse Dnmt1 promoter in vitro. A quantity of 20 ng of mouse Dnmt1 promoter (the region from − 554 to − 294 bp relative to TSS) was added to samples containing different amounts of Oct4 in 1 × binding buffer as described in the Methods section. The images were taken using an alpha gel imaging system. Lanes: (1) DL2000 markers; (2) 20 ng mouse Dnmt1 promoter; (3–9) 20 ng mouse Dnmt1 promoter interacting with increasing amounts (95, 190, 380, 570, 760, 950 and 1140 ng) of mouse Oct4 protein; (10, 11) 40 ng Oct4 protein without any mouse Dnmt1 promoter. A – Image of the EMSA gel stained with SYBR Green EMSA DNA stain to show DNA. B – The same gel stained with SYPRO Ruby EMSA protein stain to show the protein. (TIF 589 kb)

Additional file 3: **Figure S3.** Dnmt1 assay results show the promoted amount of Dnmt1 in NCI-H157 cells when overexpressed with mouse Oct4. A – Illustrated standard curve generated with Dnmt1 Standard. B and C – The final OD (equal to the 450 nm OD minus the 655 nm OD) and the amount of Dnmt1 were enhanced in the Oct4^+^ group when compared with the control (Oct4^−^ group). The results are presented as the means ± SD. The final values of (OD 450 nm – 650) from triplicate transfected samples were measured using a microplate reader. * and **Statistically significant difference of the comparisons with the negative control as determined with Student’s *t*-test at *p* < 0.05 and 0.01. (TIF 576 kb)

Additional file 4: **Table S1.** List of primer sequences used in real-time PCR, promoter analyses and ChIP assay. (DOCX 12 kb)

## Abbreviations

ChIP: Chromatin immunoprecipitation
Dnmt1: DNA (cytosine-5)-methyltransferase 1
EMSA: Electrophoretic mobility shift assay
ES cell: Embryonic stem cell
Klf4: Kruppel-like factor 4
Oct4: Octamer-binding transcription factor 4
RNAi: RNA interference
Sox2: (sex determining region Y)-box 2
