# Supplementary figures and images for: Oct4 regulates DNA methyltransferase 1 transcription by direct binding of the regulatory element
Source: Cell Mol Biol Lett. 2018 Aug 16;23:39. doi: 10.1186/s11658-018-0104-2 (PMC6097287; doi:10.1186/s11658-018-0104-2)

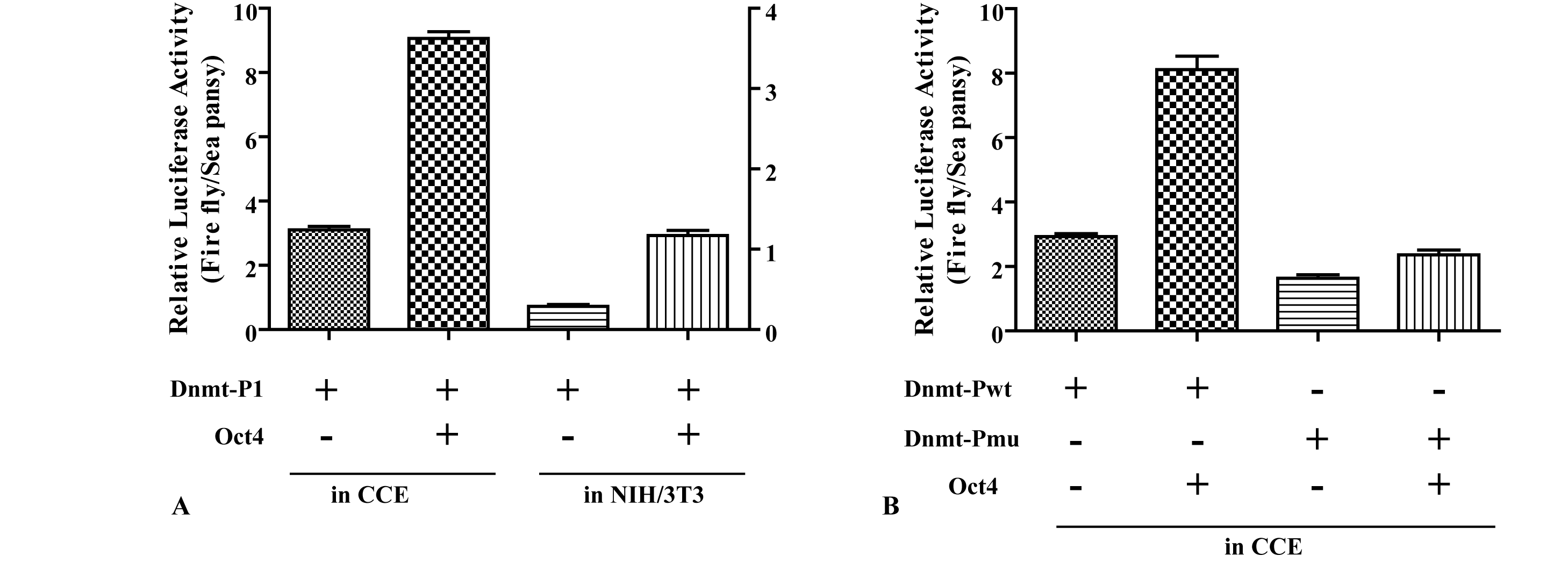

Supplement: Supplementary file 1 — Figure S1. Promoter analysis of mouse Dnmt1 in NIH3T3 and CCE cells using the luciferase assay. A – The promoter activity of mouse Dnmt1 in NIH3T3 and CCE cells. Oct4-pcDNA3.1 (100 ng) plasmid was co-transfected with Dnmt1-P1 into NIH3T3 and CCE cells. B – The promoter activity of mouse Dnmt1-Pwt and -Pmu in CCE cells. Oct4-pcDNA3.1 (100 ng) plasmid was co-transfected with Dnmt1-Pwt and -Pmu into CCE cells. The total amount of the transfected plasmid, including the pRL-TK control vector (100 ng/well), was adjusted to 1.0 μg with pcDNA3.1 empty vectors. Firefly and Renilla luciferase activities were measured 48 h after the transfection. The relative luciferase activity was calculated by dividing the activity of firefly luciferase by the activity of Renilla luciferase. The data are presented as the means ± SD for triplicate transfections. (TIF 306 kb) [file 11658_2018_104_MOESM1_ESM.tif]

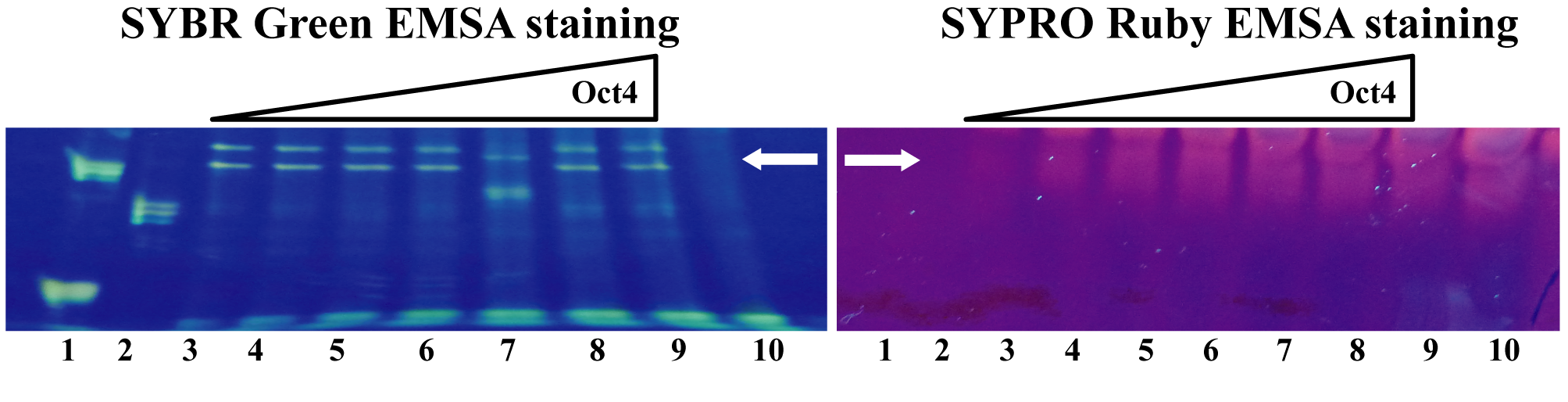

Supplement: Supplementary file 2 — Figure S2. The results of the sensitive two-color EMSA assay showed direct binding of Oct4 to the mouse Dnmt1 promoter in vitro. A quantity of 20 ng of mouse Dnmt1 promoter (the region from − 554 to − 294 bp relative to TSS) was added to samples containing different amounts of Oct4 in 1 × binding buffer as described in the Methods section. The images were taken using an alpha gel imaging system. Lanes: (1) DL2000 markers; (2) 20 ng mouse Dnmt1 promoter; (3–9) 20 ng mouse Dnmt1 promoter interacting with increasing amounts (95, 190, 380, 570, 760, 950 and 1140 ng) of mouse Oct4 protein; (10, 11) 40 ng Oct4 protein without any mouse Dnmt1 promoter. A – Image of the EMSA gel stained with SYBR Green EMSA DNA stain to show DNA. B – The same gel stained with SYPRO Ruby EMSA protein stain to show the protein. (TIF 589 kb) [file 11658_2018_104_MOESM2_ESM.tif]

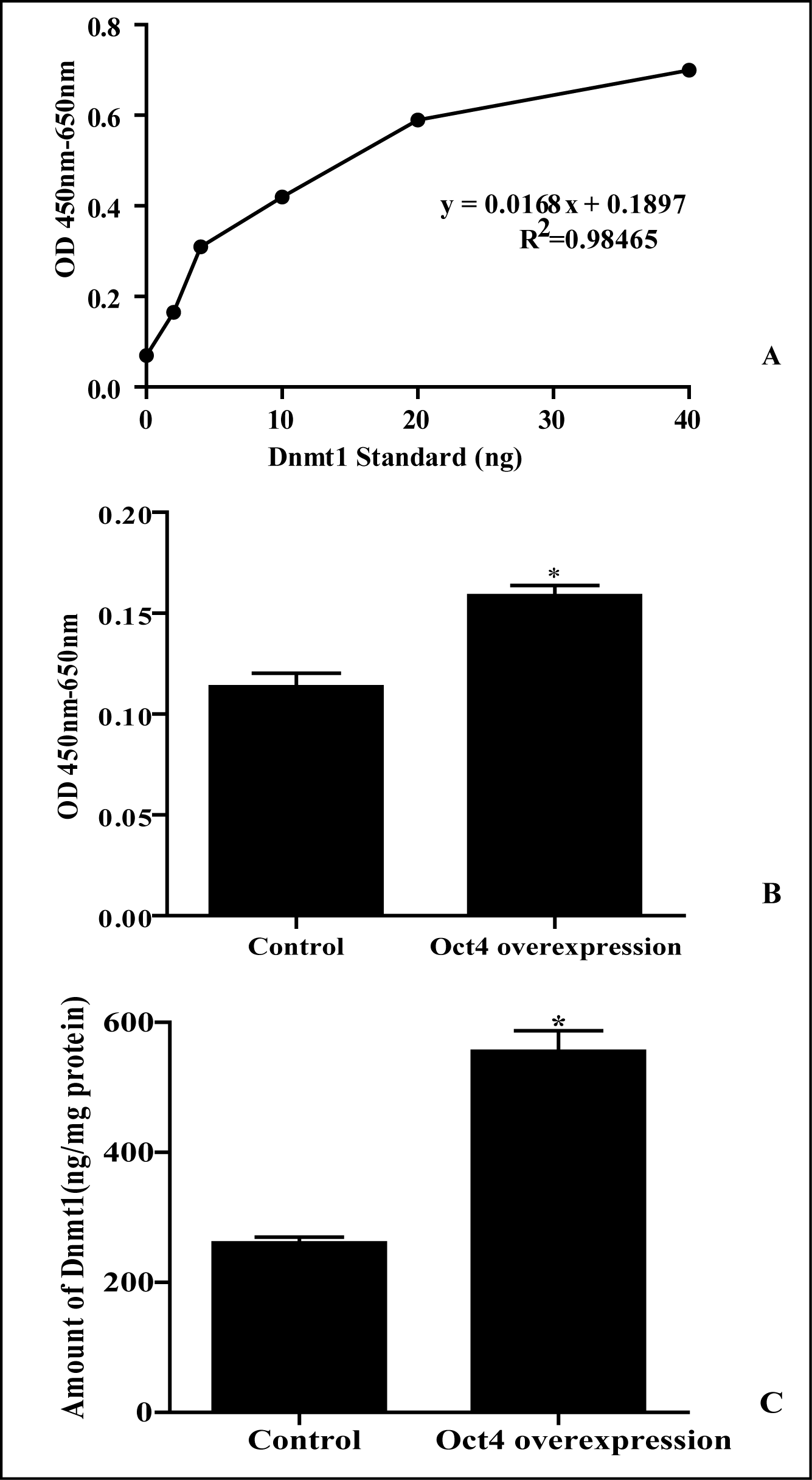

Supplement: Supplementary file 3 — Figure S3. Dnmt1 assay results show the promoted amount of Dnmt1 in NCI-H157 cells when overexpressed with mouse Oct4. A – Illustrated standard curve generated with Dnmt1 Standard. B and C – The final OD (equal to the 450 nm OD minus the 655 nm OD) and the amount of Dnmt1 were enhanced in the Oct4+ group when compared with the control (Oct4− group). The results are presented as the means ± SD. The final values of (OD 450 nm – 650) from triplicate transfected samples were measured using a microplate reader. * and **Statistically significant difference of the comparisons with the negative control as determined with Student’s t-test at p < 0.05 and 0.01. (TIF 576 kb) [file 11658_2018_104_MOESM3_ESM.tif]
