# Supplementary material for: Oct4 regulates DNA methyltransferase 1 transcription by direct binding of the regulatory element
Source: Cell Mol Biol Lett. 2018 Aug 16;23:39. doi: 10.1186/s11658-018-0104-2 (PMC6097287; doi:10.1186/s11658-018-0104-2)
Supplement: Supplementary file 4 — Table S1. List of primer sequences used in real-time PCR, promoter analyses and ChIP assay. (DOCX 12 kb) [file 11658_2018_104_MOESM4_ESM.docx]

**Table S1**. List of primer sequences used in real-time PCR, promoter analyses and ChIP assay.

| **Primer** | **Sequence** | **Purpose** |
| --- | --- | --- |
| Dnmt1-F | CCTAGTTCCGTGGCTACGAGGAGAA | For real-time PCR |
| Dnmt1-R | TCTCTCTCCTCTGCAGCCGACTCA |  |
| Gapdh-F | ATTCAACGGCACAGTCAAGG |  |
| Gapdh-R | GGTCCTCAGTGTAGCCCAAGA |  |
| Dnmt1-P1F | CGACGCGTTATACTACTTCATTGG | For promoter assay |
| Dnmt1-P2F | CGACGCGTTTAGACACTACAGAACC |  |
| Dnmt1-P3F | CGACGCGTGCTGACCTCAAACTGAGA |  |
| Dnmt1-P4F  Dnmt1-Pwt  Dnmt1-Pmu | CGACGCGTAAGAGGCTGTAGGACC  ‍CGACGCGTCACAGAGATCCTCTGGCTTTTGCAT  TCTGAGTGCT  CGACGCGTCACAGAGATCCTCTGGaccccatgc  TCTGAGTGCT |  |
| Dnmt1-PR | CCCAAGCTT GCGCAAGCGGAAGCAGCA |  |
| Oct4-F | CGGAATTCGCCACCATGGCTGGACACCTGG | For pcDNA3.1 expression plasmid |
| Oct4-R | CCGCTCGAGTCAGTTTGAATGCATG |  |
| Dnmt1-CF | GCCACCACCGCCTACCTTT | For ChIP |
| Dnmt1-CR | GTCCTACAGCCTCTTCTGGC |  |

Underlined bases show the restriction sites.
